# Microbiome definition re-visited: old concepts and new challenges

**DOI:** 10.1186/s40168-020-00875-0

**Published:** 2020-06-30

**Authors:** Gabriele Berg, Daria Rybakova, Doreen Fischer, Tomislav Cernava, Marie-Christine Champomier Vergès, Trevor Charles, Xiaoyulong Chen, Luca Cocolin, Kellye Eversole, Gema Herrero Corral, Maria Kazou, Linda Kinkel, Lene Lange, Nelson Lima, Alexander Loy, James A. Macklin, Emmanuelle Maguin, Tim Mauchline, Ryan McClure, Birgit Mitter, Matthew Ryan, Inga Sarand, Hauke Smidt, Bettina Schelkle, Hugo Roume, G. Seghal Kiran, Joseph Selvin, Rafael Soares Correa de Souza, Leo van Overbeek, Brajesh K. Singh, Michael Wagner, Aaron Walsh, Angela Sessitsch, Michael Schloter

**Affiliations:** 1 Environmental Biotechnology, Graz University of Technology, Graz, Austria; 2 Helmholtz Zentrum München, Oberschleissheim, Germany; 3 MICALIS, INRA, AgroParisTech, Université Paris-Saclay, 78350 Jouy-en-Josas, France; 4 Waterloo Centre for Microbial Research, University of Waterloo, 200 University Avenue West, Waterloo, ON N2L 3G1 Canada; 5 Metagenom Bio, 550 Parkside Drive, Unit A9, Waterloo, ON N2L 5 V4 Canada; 6 Guizhou Provincial Key Laboratory for Agricultural Pest Management of the Mountainous Region, Guizhou University, Guiyang, 550025 Guizhou China; 7 European Food Information Council, Brussels, Belgium; 8 International Alliance for Phytobiomes Research, Summit, Lee, MO ’s USA; 9 MICA, INRA, 78350 Jouy-en-Josas, France; 10 Laboratory of Dairy Research, Department of Food Science and Human Nutrition, Agricultural University of Athens, Athens, Greece; 11 Department of Plant Pathology, University of Minnesota, St. Paul, MN 55108 USA; 12 BioEconomy, Research, & Advisory, Valby, Denmark; 13 CEB-Centre of Biological Engineering, University of Minho, Campus de Gualtar, 4710-057 Braga, Portugal; 14 Department of Microbial Ecology and Ecosystem Science, University of Vienna, Vienna, Austria; 15 Agriculture and Agri-Food Canada, Ottawa, Canada; 16 Sustainable Agriculture Sciences, Rothamsted Research, Harpenden, UK; 17 Biological Sciences Division, Pacific Northwest National Laboratory, Richland, WA 99352 USA; 18 Bioresources Unit, AIT Austrian Institute of Technology, Tulln, Austria; 19 CABI, Bakeham Lane, Egham, Surrey, TW20 9TY UK; 20 Department of Chemistry and Biotechnology, Tallinn University of Technology, Tallinn, Estonia; 21 Laboratory of Microbiology, Wageningen University & Research, Wageningen, the Netherlands; 22 MGP, INRA, 78350 Jouy-en-Josas, France; 23 Dept of Food Science and Technology, Pondicherry University, Puducherry, India; 24 Department of Microbiology, Pondicherry University, Puducherry, India; 25 Genomics for Climate Change Research Center (GCCRC), Universidade Estadual de Campinas (UNICAMP), Campinas, SP Brazil; 26 Hawkesbury Institute for the Environment, Western Sydney University, Penrith, NSW Australia; 27 Global Centre for Land-Based Innovation, Western Sydney University, Penrith, NSW Australia; 28 Teagasc Food Research Centre, Moorepark, Fermoy, Co. Cork, Ireland

## Abstract

The field of microbiome research has evolved rapidly over the past few decades and has become a topic of great scientific and public interest. As a result of this rapid growth in interest covering different fields, we are lacking a clear commonly agreed definition of the term “microbiome.” Moreover, a consensus on best practices in microbiome research is missing. Recently, a panel of international experts discussed the current gaps in the frame of the European-funded MicrobiomeSupport project. The meeting brought together about 40 leaders from diverse microbiome areas, while more than a hundred experts from all over the world took part in an online survey accompanying the workshop. This article excerpts the outcomes of the workshop and the corresponding online survey embedded in a short historical introduction and future outlook. We propose a definition of microbiome based on the compact, clear, and comprehensive description of the term provided by Whipps et al. in 1988, amended with a set of novel recommendations considering the latest technological developments and research findings. We clearly separate the terms microbiome and microbiota and provide a comprehensive discussion considering the composition of microbiota, the heterogeneity and dynamics of microbiomes in time and space, the stability and resilience of microbial networks, the definition of core microbiomes, and functionally relevant keystone species as well as co-evolutionary principles of microbe-host and inter-species interactions within the microbiome. These broad definitions together with the suggested unifying concepts will help to improve standardization of microbiome studies in the future, and could be the starting point for an integrated assessment of data resulting in a more rapid transfer of knowledge from basic science into practice. Furthermore, microbiome standards are important for solving new challenges associated with anthropogenic-driven changes in the field of planetary health, for which the understanding of microbiomes might play a key role.

Video Abstract

Video Abstract

## Introduction

Improving our knowledge of microbiomes has become a popular topic over the past two decades not only in the scientific community, but also among the general public, especially as an area of great promise for new medical treatments. The human microbiome is now even considered to be our “last organ” [1]. Research on the human microbiome has advanced from a fledgling field to a flourishing area of medical research with more than US$1.7 billion being spent only over the past decade alone [2]. Promising results from microbiome research also boosted the whole “microbiome market” and private investment into companies and startups (www.global-engage.com). In addition to human health, microbiome research provides a foundation for a much broader scope of applications [3]. Advances in engineering of environmental microbiomes will replace toxic chemicals in agri-, horti-, and aquaculture in the future and stimulate a more sustainable use of environmental resources, as well as improve our food processing [4–9]. Agricultural products based on the microbiota are one of the fastest growing sectors in agronomy with a Compound Annual Growth Rate (CAGR) of 15–18% and a predicted value of over 10 billion US dollars by 2025 [10]. Moreover, microbiome research may provide solutions on how humans and other life forms on Earth can contribute to withstand one of our main problems: the anthropogenic-driven climate change [11].

Historically, the field of microbiome research has emerged from environmental microbiome research (microbial ecology) and provides an interdisciplinary platform for many fields, e.g., agriculture, food science, biotechnology, bioeconomy, mathematics (informatics, statistics, modeling), plant pathology, and especially human medicine. The new field has already delivered novel and important concepts for describing host-microbial interactions such as the holobiont theory or meta-organism concept [12–14]. Further, principles of coevolution, co-selection, and stress response within microbiomes go far beyond the traditional scope of these concepts [15, 16]. Stegen et al. [17] suggest a unified conceptual framework for prediction and control of microbiomes. Significantly, there has been a fundamental paradigm shift in our understanding of microorganisms and it is now accepted that all eukaryotes are meta-organisms and must be considered together with their microbiota as an inseparable functional unit [18]. This concept also considers the fact that pathogens represent only a tiny fraction of microorganisms; diversity loss can result in a so-called “dysbiosis” that describes the altered composition of microbes, which has a cascading impact on the immune system and offers an advantage for emergence and outbreak of pathogens [19–21].

Considering the fundamental nature of microbial life and diversity in relation to larger organisms and vice versa, scientists are calling for a rethinking of the role of microorganisms [22, 23]. In spite of the substantial popularity of microbiome research across diverse fields, this extremely fast-growing discipline faces a variety of challenges. The lack of data standardization as a matter of continuous development of new techniques and equipment, as well as the urgent need for better coordination and collaboration across the field of microbiome research, was recently listed as the most important challenges facing microbiome researchers [2]. Furthermore, a consistent criticism has been that microbiome research is more driven by methods than by hypotheses or concepts [24]. To provide mechanistic insights in microbiota functioning is not only important for the human microbiome, it affects all areas in this research field. However, a clear or consensus definition of “microbiome” among researchers from diverse fields remains debatable [25].

The main goal of this article is to overcome the obstacles faced in microbiome research by proposing an explicit definition for the term microbiome and building a common ground for microbiome researchers. Our definition builds on previous definitions with amendments covering areas that are still emerging and thus exposed to fluctuations. The article is based on discussions in the frame of a workshop which took place on 6 March 2019 in Tulln (Austria) as part of the MicrobiomeSupport project [26], which aims to establish international research standards. The workshop brought together leading microbiome researchers from academic, governmental, and industry groups representing diverse areas of expertise. Prior to the workshop, an online survey was sent to address critical questions on the definition of the term microbiome as well as challenges in microbiome research and development to which more than a hundred experts from all over the world responded. The outcomes of the survey and subsequent workshop discussions form the foundation for the proposed definition of the term microbiome, and the set of amendments containing the rules and baselines for microbiome research is described here. Moreover, this article provides an overview on the historical development of microbial research and shows how this development shaped the various existing microbiome definitions. We provide a recommendation based on an existing definition of the term microbiome and lay out principles for this choice. As microbiome research is driven by highly sophisticated technology development and grapples with an enormous quantity of complex data, we also discuss the definition of microbiome in relation to ongoing technical developments. In addition, we provide specific recommendations to stimulate microbiome researchers to generate more detailed information related to microbiome functionality, increase the capacities to integrate and compare data obtained in different studies, and facilitate a more efficient transfer of results from basic science to application.

## From microorganisms to microbiomes: a historical overview

**Fig. 1:**
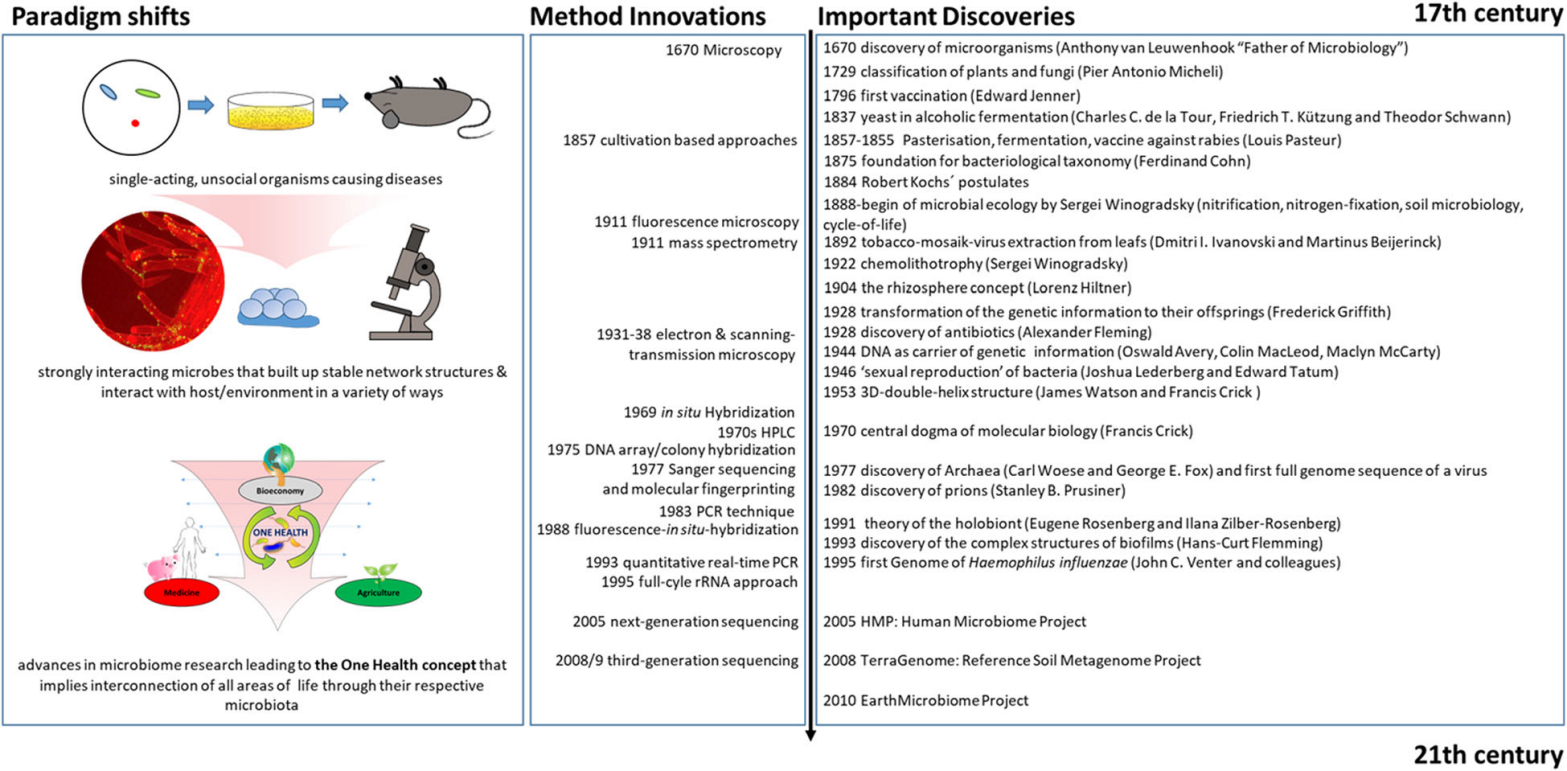
The history of microbiome research from seventieth century until our days, highlighting the shift of the paradigm from microbes as unsocial organisms causing diseases to the holistic view of microorganisms being the center of the One Health Concept: positively interconnecting all areas of our lives. The list of the literature used for this figure can be found in the Supplemental File 1

Taking major historical developments into account is important to understand how microbiome research has manifested itself as core discipline in modern life. The field of microbiome research originated in microbiology and started back in the seventieth century. Research progress has often been driven by the development of new techniques and equipment. Interestingly, many technological inventions have boosted microbiological research in such a manner and caused paradigm shifts in our understanding of health and disease (Fig. 1). Since infectious diseases have affected human populations throughout most of history, medical microbiology was the earliest focus of research and public interest. Additionally, food microbiology is an old field of empirical applications. The development of the first microscopes allowed the discovery of a new, unknown world and led to the identification of microorganisms. Access to the previously invisible world opened the eyes and the minds of the researchers of the seventieth century. Antonie van Leeuwenhoek investigated diverse bacteria of various shapes, fungi, and protozoa, which he called animalcules, mainly from water, mud, and dental plaque samples, and discovered biofilms as a first indication of microorganisms interacting within complex communities. Robert Koch’s explanation of the origin of human and animal diseases as a consequence of microbial infection and development of the concept of pathogenicity was an important milestone in microbiology. These findings shifted the focus of the research community and the public on the role of microorganisms as disease-forming agents that needed to be eliminated. However, comprehensive research over the past century has shown that only a small proportion of microorganisms are associated with disease or pathogenicity; the overwhelming majority of microbes are essential for ecosystem functioning and known for beneficial interactions with other microbes as well as macroorganisms. At the end of the ninetieth century, microbial ecology started with the pioneering work by Martinus W. Beijerinck and Sergei N. Winogradski. The newly established science of environmental microbiology resulted in another paradigm shift: microorganisms are everywhere in natural environments, often associated with hosts and, for the first time, beneficial effects on their hosts were reported [27, 28]. Subsequently, the concept that microorganisms exist as single cells began to change as it became increasingly obvious that microbes occur within complex assemblages in which species interactions and communication are critical to population dynamics and functional activities [29]. Discovery of DNA, the development of sequencing technologies, PCR, and cloning techniques enabled the investigation of microbial communities using cultivation-independent, DNA and RNA-based approaches [30]. A further important step was the introduction of phylogenetic markers such as the 16S rRNA gene for microbial community analysis by Carl Woese and George E. Fox in 1977 [31]. Today, we are able to barcode bacteria, archaea, fungi, algae, and protists in their natural habitats, e.g., by targeting their 16S and 18S rRNA genes, internal transcribed spacer (ITS), or, alternatively, specific functional regions of genes coding for specific enzymes [32–34]. Another major paradigm shift was initiated at the beginning of this century and continues through today, as new sequencing technologies and accumulated sequence data have highlighted both the ubiquity of microbial communities in association within higher organisms and the critical roles of microbes in human, animal, and plant health [35]. These new possibilities have revolutionized microbial ecology, because the analysis of genomes and metagenomes in a high-throughput manner provides efficient methods for addressing the functional potential of individual microorganisms as well as of whole communities in their natural habitats [36, 37]. Multi-omics technologies including metatranscriptome, metaproteome, or metabolome approaches now provide detailed information on microbial activities in the environment. Based on the rich foundation of data, the cultivation of microbes, which was often ignored or underestimated in the last 30 years, has gained new importance, and high throughput culturomics is now an important part of the toolbox to study microbiomes. The high potential and enormous power of combining multiple “omics” techniques to analyze host-microbe interactions are highlighted in several reviews [17, 38].

## Defining the microbiome—current definitions and gaps

Microbial communities have commonly been defined as the collection of microorganisms living together. More specifically, microbial communities are defined as multi-species assemblages, in which (micro) organisms interact with each other in a contiguous environment [39]. In 1988, Whipps and colleagues working on the ecology of rhizosphere microorganisms provided the first definition of the term microbiome [40]. They described the “microbiome” as a combination of the words “micro” and “biome”, naming a “characteristic microbial community” in a “reasonably well-defined habitat which has distinct physio-chemical properties” as their “theatre of activity” (Table 1). This definition represents a substantial advancement of the definition of a microbial community, as it defines a microbial community with distinct properties and functions and its interactions with its environment, resulting in the formation of specific ecological niches. However, there are many other microbiome definitions that have been published in the last few decades. The currently most cited definition by Lederberg [42] describes microbiomes within an ecological context, as a community of commensal, symbiotic, and pathogenic microorganisms within a body space or other environment. Marchesi and Ravel focused in their definition on the genomes and microbial (and viral) gene expression patterns and proteomes in a given environment and its prevailing biotic and abiotic conditions [25]. All these definitions imply that general concepts of macro-ecology could be easily applied to microbe-microbe as well as to microbe-host interactions. However, the extent to which these concepts, developed for macro-eukaryotes, can be applied to prokaryotes with their different lifestyles regarding dormancy, variation of phenotype, and horizontal gene transfer [52] as well as to micro-eukaryotes that is not quite clear. This raises the challenge of considering an entirely novel body of conceptual ecology models and theory for microbiome ecology, particularly in relation to the diverse hierarchies of interactions of microbes with one another and with the host biotic and abiotic environments. Many current definitions fail to capture this complexity and describe the term microbiome as encompassing the genomes of microorganisms only (Table 1). Merriam Webster publishing platform, for example, suggests two microbiome definitions: one describing the metagenome and the other is the community of microorganisms, yet still fails to capture the host and the environment as an integral ecological component of the microbiome, rather than an independent entity.

**Table 1:** Microbiome definitions

**Ecological definitions** Definitions based on ecology describe the microbiome following the concepts derived from the ecology of multicellular organisms. The main issue here is that the theories from the macro-ecology do not always fit the rules in the microbial world. “A convenient ecological framework in which to examine biocontrol systems is that of the microbiome. This may be defined as a characteristic microbial community occupying a reasonably well-defined habitat which has distinct physio-chemical properties. The term thus not only refers to the microorganisms involved but also encompasses their theatre of activity ”[40]. “…This term refers to the entire habitat, including the microorganisms (bacteria, archaea, lower and higher eurkaryotes, and viruses), their genomes (i.e., genes), and the surrounding environmental conditions. This definition is based on that of “biome,” the biotic and abiotic factors of given environments. Others in the field limit the definition of microbiome to the collection of genes and genomes of members of a microbiota. It is argued that this is the definition of metagenome, which combined with the environment constitutes the microbiome. The microbiome is characterized by the application of one or combinations of metagenomics, metabonomics, metatranscriptomics, and metaproteomics combined with clinical or environmental metadata” [25]. “others use the term microbiome to mean all the microbes of a community, and in particular, for the plant microbiome, those microbial communities associated with the plant which can live, thrive, and interact with different tissues such as roots, shoots, leaves, flowers, and seeds” (from Orozco-Mosqueda et al. [41]). “Ecological community of commensal, symbiotic and pathogenic microorganisms within a body space or other environment” [42].	
**Organisms/host-dependent definitions** The host-dependent definitions are based on the microbial interactions with the host. The main gaps here concern the question whether the microbial-host interaction data gained from one host can be transferred to another. The understanding of coevolution and selection in the host-dependent definitions is also underrepresented. “A community of microorganisms (such as bacteria, fungi, and viruses) that inhabit a particular environment and especially the collection of microorganisms living in or on the human body” [43]. “Human Microbiome Project (HMP): [...] The Human Microbiome is the collection of all the microorganisms living in association with the human body. These communities consist of a variety of microorganisms including eukaryotes, archaea, bacteria and viruses” [44].	
**Genomic/ method-driven definitions** There is a variety of microbiome definitions available that are driven by the methods applied. Mostly, these definitions rely on DNA sequence-based analysis and describe microbiome as a collective genome of microorganisms in a specific environment. The main bottleneck here is that every new available technology will result in a need for a new definition. “The collective genomes of microorganisms inhabiting a particular environment and especially the human body” [43]. “The microbiome comprises all of the genetic material within a microbiota (the entire collection of microorganisms in a specific niche, such as the human gut). This can also be referred to as the metagenome of the microbiota” [45]. “Microbiome is a term that describes the genome of all the microorganisms, symbiotic and pathogenic, living in and on all vertebrates. The gut microbiome is comprised of the collective genome of microbes inhabiting the gut including bacteria, archaea, viruses, and fungi” [46]. “Different approaches to define the population provide different information. a | Microbiota: 16S rRNA surveys are used to taxonomically identify the microorganisms in the environment. b | Metagenome: the genes and genomes of the microbiota, including plasmids, highlighting the genetic potential of the population. c | Microbiome: the genes and genomes of the microbiota, as well as the products of the microbiota and the host environment” [47]. “Totality of genomes of a microbiota. Often used to describe the entity of microbial traits (=functions) encoded by a microbiota.” [48]	
**Combined definitions** There are some microbiome definitions available that fit several categories with their advantages and disadvantages. “A microbiome is the ecological community of commensal, symbiotic, and pathogenic microorganisms that literally share our body space” [49]. “The microbiome is the sum of the microbes and their genomic elements in a particular environment” [50]. “The genes and genomes of the microbiota, as well as the products of the microbiota and the host environment” [51].	

A revised conceptual framework will allow us to move from cataloguing microorganisms to a more holistic view on microbial functioning and interaction with its environment. This, however, will require substantially more interdisciplinary interaction between scientists working across disparate fields [2]. The variety of perspectives on the term microbiome were a central part of discussion within the MicrobiomeSupport workshop. Based on the responses obtained in the online survey and workshop discussions, participants concluded that the original definition of Whipps et al. [40] is still the most comprehensive one that captures the complexity of the microbiome and the diverse facets of its ecology and evolutionary biology. The workshop participants discussed a number of critical points, resulting in recommendations for clarifications and amendments to the original Whipps and coworkers’ definition. These amendments address (1) members of the microbiome, (2) interactions among members of the microbiome and within existing microbial networks, (3) spatial and temporal characteristics of microbiomes in their environment, (4) the core microbiota, (5) moving from functional predictions to phenotypes of species, and (6) microbiome—host or environmental interactions and coevolution. Below, we discuss these aspects in detail.

### Members of the microbiome

**Fig. 2:**
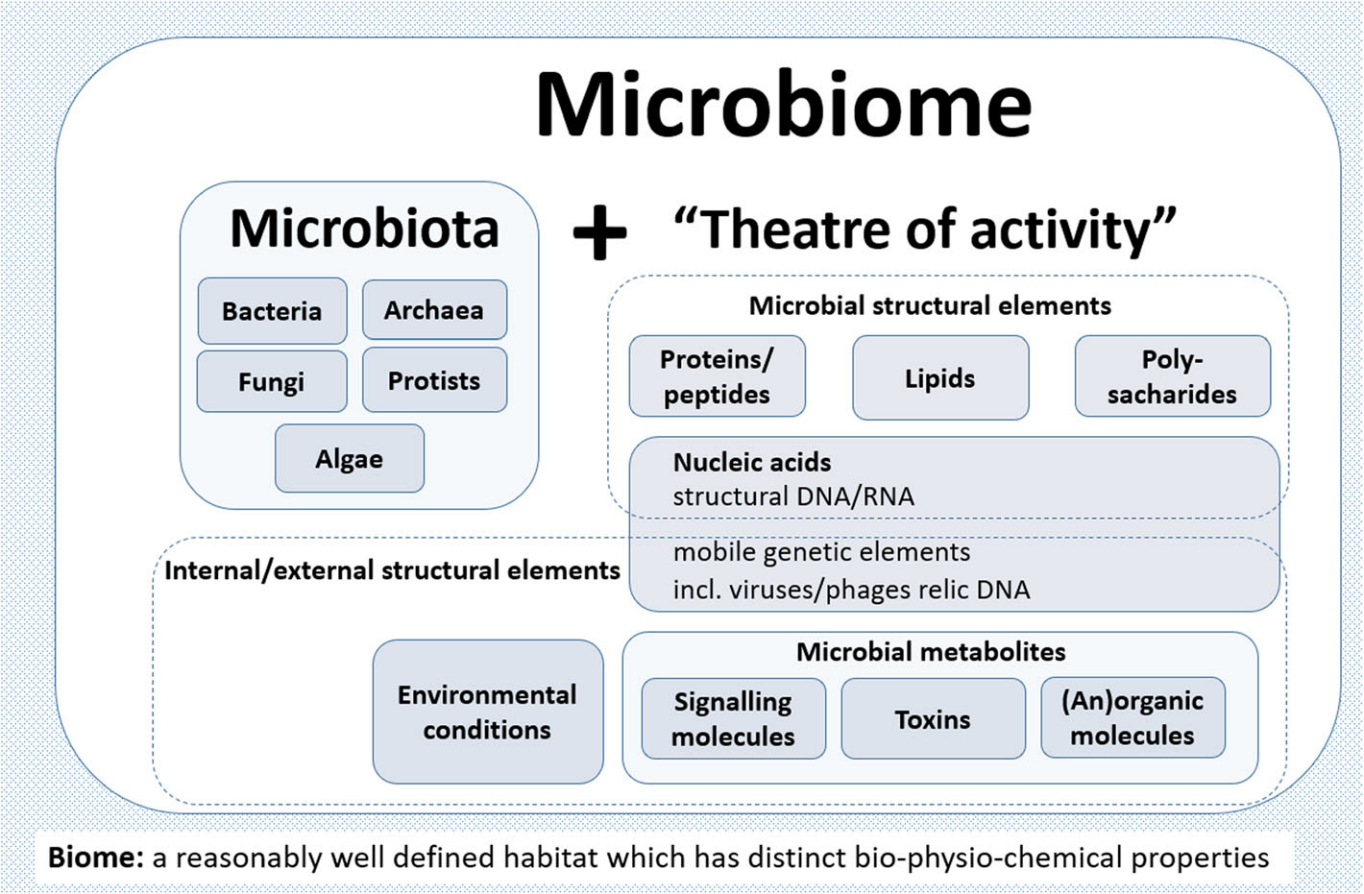
A schematic highlighting the composition of the term microbiome containing both the microbiota (community of microorganisms) and their “theatre of activity” (structural elements, metabolites/signal molecules, and the surrounding environmental conditions)

The microbiota comprises all living members forming the microbiome. Etymology and differences of both terms are explained in Table 2. Bacteria, archaea, fungi, algae, and small protists should be considered as members of the microbiome [25]; most microbiome researchers agree with this definition. The integration of phages, viruses, plasmids, and mobile genetic elements is one of the most controversial points in the definition of the microbiome. This was also confirmed by the participants’ comments in the “microbiome definition” online survey. The answers of the survey participants to the question whether viruses and phages should be a part of the microbiome provided no clear answer and stretched from “in any case” to “definitely no.” There is also no clear consensus as to whether extracellular DNA derived from dead cells (so-called “relic DNA”) belongs to the microbiome. Relic DNA can comprise up to 40% of the sequenced DNA in soil [53] and was up to 33% of the total bacterial DNA on average in a broader analysis of habitats with the highest proportion of 80% in some samples [54]. Interestingly, despite its omnipresence and abundance, relic DNA had a minimal effect on estimates of taxonomic and phylogenetic diversity [54]. When it comes to the use of specific terms, a clear differentiation between microbiome and microbiota helps to avoid the controversy concerning the members of a microbiome (Fig. 2). Microbiota is usually defined as the assemblage of *living* microorganisms present in a defined environment [25]. As phages, viruses, plasmids, prions, viroids, and free DNA are usually not considered as living microorganisms [55], they do not belong to the microbiota. The term microbiome, as it was originally postulated by Whipps and coworkers [40], includes not only the community of the microorganisms, but also their “theatre of activity.” The latter involves the whole spectrum of molecules produced by the microorganisms, including their structural elements (nucleic acids, proteins, lipids, polysaccharides), metabolites (signaling molecules, toxins, organic, and inorganic molecules), and molecules produced by coexisting hosts and structured by the surrounding environmental conditions. Therefore, all mobile genetic elements, such as phages, viruses, and “relic” and extracellular DNA, should be included in the term microbiome, but are not a part of microbiota (Fig. 2). Moreover, in this regard, it is important to consider methodological aspects to differentiate DNA from living organisms and their environment (see the chapter technical standards). The term microbiome is also sometimes confused with the metagenome. Metagenome is, however, clearly defined as a collection of genomes and genes from the members of a microbiota [25].

**Table 2:** Microbiome/microbiota etymology

**Microbiome** The words “micro” and “biome” are of Ancient Greek origin. “Micro” (μικρος) means small, while the term “biome” is composed of the Greek word bíos (βιος, life) and modified by the ending “ome” (Anglicization of Greek). **Microbiota** The words “micro” and “biota” are also of Ancient Greek origin. It is a combination of “Micro” (μικρος, small), with the term “biota” (βιοτα), which means the living organisms of an ecosystem or a particular area.

Microbiome studies sometimes focus on the behavior of a specific group of microbiota (Fig. 2), generally in relation to or justified by a clear hypothesis. Despite more and more terms like “bacteriome,” “archaeome,” “mycobiome,” or “virome” have started to appear in the scientific literature, and these terms do not refer to biomes (a regional ecosystem with a distinct assemblage of (micro) organisms, and physical environment often reflecting a certain climate and soil) as the microbiome itself. Consequently, it would be better to use the original terms (bacterial, archaeal, or fungal community). In contrast to the microbiota, which can be studied separately, the microbiome is always composed by all members, which interact with each other, live in the same habitat, and form their ecological niche together. The well-established term “virome” is derived from “virus” and “genome” and is used to describe viral shotgun metagenomes consisting of a collection of nucleic acids associated with a particular ecosystem or holobiont [56]. However, also here “viral metagenomes” can be suggested as semantically and scientifically better terms.

There is also a question of at what resolution each of the microbiome members should be studied. For eukaryotes, in most cases, the “reproductive unit” is an adequate level, where dynamics of organisms are measured [57, 58], though the species definition continues to be debated [59]. For prokaryotes, however, such a definition based on reproduction does not exist: current species definitions are based on DNA homologies between organisms such as the “more than 70% DNA-similarity” revealed by DNA-DNA hybridization [58], or by the average nucleotide identity as recommended by Goris and colleagues [60]. Similarly to eukaryotes, the microbial strains or ecotypes are the basis of taxonomy and functionality. The stability of the defined strain is here, the most critically discussed issue mostly due to the frequent occurrence of horizontal gene transfer (HGT). The latter is induced by the shift of the mobile genetic elements such as plasmids, bacteriophages, and transposons from one strain to another, resulting in inevitable genome changes and strongly affecting the strain stability. Neglecting the strain level may, however, result in a misinterpretation of the data due to essential functional differences between microbial strains [61]. Defining ecologically meaningful populations among microbial strains is important for identifying their roles in environmental and host-associated microbiomes. Recently, a novel metric of recent gene flow was introduced as a solution, which identifies congruent genetic and ecological units separated by strong gene flow discontinuities from their next of kin [47].

### Microbial networks and interactions

**Fig. 3:**
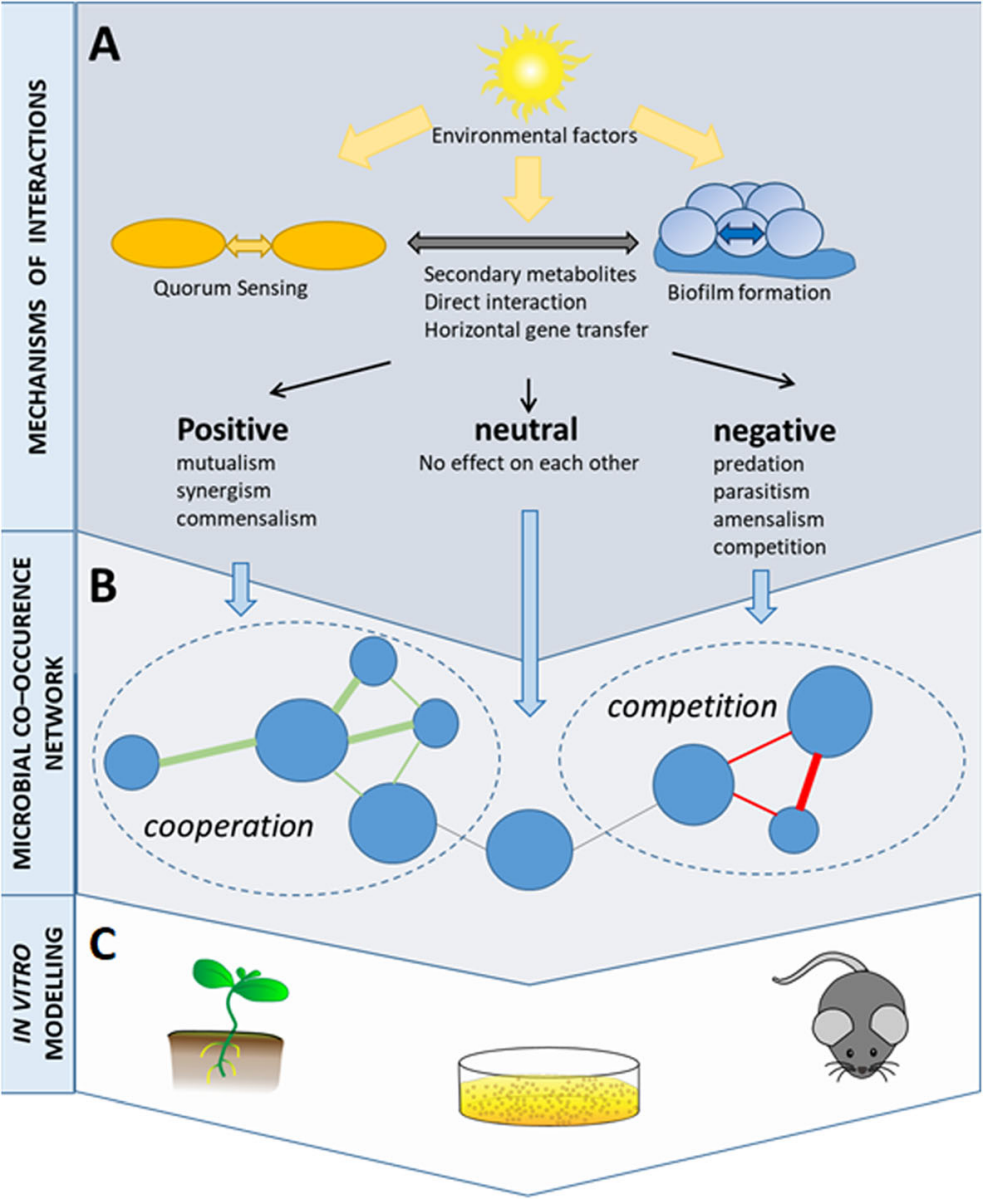
Microbial interactions visualized through microbial co-occurrence networks. **a** Microbial interactions are influenced by environmental factors and are separated into positive, neutral, and negative interactions types. **b** Microbial co-occurrence and co-exclusion networks help visualizing microbial interactions. In such networks, nodes usually represent taxa of microorganisms, and edges represent statistically significant associations between nodes. Green edges usually stay for positive interactions, while red edges visualize negative interactions between the microorganisms. **c** Testing of the hypotheses resulted from the network analyses in relevant model systems is required for a comprehensive study of microbial interactions

Microbes interact with one another, and these symbiotic interactions have diverse consequences for microbial fitness, population dynamics, and functional capacities within the microbiome [62]. These interactions can either be between microorganisms of the same species or between different species, genera, families, and domains of life. The interactive patterns within these webs may be positive (mutualism, synergism, or commensalism), negative (amensalism [including predation, parasitism, antagonism, or competition]), or neutral—where there is no (or no observed) effect on the functional capacities or fitness of interacting species (Fig. 3a). Microbial life strategy concepts (i.e., copio- and oligotrophic strategists and competitor–stress tolerator–ruderals framework) can influence outcomes of interactions [63]. For example, microorganisms competing for the same source can also benefit from each other when competing for the same compound at different trophic levels. Stability of a complex microbial ecosystem depends on trophic interactions for the same substrate at different concentration levels. It is important to highlight that microbial social adaptations in nature are so far understudied. Here, molecular markers can provide insight into social adaptations by supporting the theories, e.g., of altruists and cheaters in native microbiomes [64].

Secondary metabolites play an essential role in mediating complex interspecies interactions and ensure survival in competitive environments. Quorum sensing (QS) induced by small molecules like n-acyl-homoserine lactones or peptides allows bacteria to control cooperative activities and adapts their phenotypes to the biotic environment, resulting, e.g., in cell-cell adhesion or biofilm formation [29, 65]. Direct Interspecies Electron Transfer (DIET) is an important mechanism for communication in most anaerobic ecosystems [66]. In addition, volatile compounds can act as long-term messengers for cross-kingdom communication over long distances [67]. Moreover, the so-called “fungal highways” serve as transportation systems for bacteria [68] as well as for water and nutrients [69] and can therefore play an important role in structuring microbial networks. Despite these examples, communication and interaction within the microbiome remain understudied and would profit from more knowledge on the metabolic interplay of all microbiome members. Here, reductionist experimental models and model microbiomes can help to identify microbes and molecular mechanisms involved in complex interactions [70].

Bioinformatic network and co-occurrence analyses give an idea about the complexity of microbial interaction patterns [71, 72] but they are not suitable to unravel the nature of these interactions (Fig. 3b). Despite this limitation, analysis of microbial networks allows researchers to identify hub species and explore the potential for diverse types of species interactions within the microbiome. In microbial co-occurrence networks, hub species are represented by nodes (Fig. 3b) that have highest degree of connections with other species [72]. Co-occurrence analyses can be also applied at different scales, e.g., co-occurrence patterns between ecosystems at the community scale, modules of co-occurring microorganisms within communities, and co-occurring pairs within modules that are nested within microbial communities [73]. They can be linked to colonization resistance, which determines the potential for allochthonous microorganisms to invade the autochthonous community, and can be considered as important tools for hypothesis generation [4, 74, 75]. The existence of specific types of microbial interactions and their consequences for population dynamics or functions, however, require testing in relevant model systems (Fig. 3c). Additionally, technical approaches, such as cross feeding experiments with stable isotopes [75] or Fluorescence in situ Hybridization and Confocal Laser Scanning Microscopy (FISH-CLSM) combined with dual culture assays [76, 77], are extremely useful for testing hypotheses generated in silico.

Microbial interactions can be an important basis for evolutionary and co-evolutionary dynamics within microbiomes [78, 79]. Communication between microbial community members gives rise to a complex landscape in which the fitness or function of a cell depends not only on the genetic potential and chemical environment of a single cell, but also on the biotic environment sensed [80, 81]. Hub species in networks are often hypothesized to serve as keystone species, a concept which has been transferred from macro-ecology into microbiome research [82]. Keystone species are suggested to play a crucial role in diverse species interactions and to have a greater impact on the performance and dynamics of an ecosystem than other species [62]. However, hub species within a co-association network do not necessarily play a role as keystone species. The characterization of the latter ones must additionally be confirmed and complemented by adequate methods [83, 84]. Hub and keystone taxa definitively need a better understanding of their function in situ [85, 86]; in addition, they can be integrated in computational methods to link micro- and macroecological questions. If keystone species can be considered as “indicator taxa”, another term, which have been defined as those taxa that are highly indicative of a particular experimental treatment or environmental condition [87] is still unclear. The concept of indicator taxa has gained much interest from the practical point of view and was transferred from macroecology into microbiome research, and is now frequently used, e.g., in studies assessing the impact of agricultural practices on microbiomes [88] or of diseases on the human microbiota [89]; as here, simple and highly standardized qPCR-based approaches can be used for analysis.

### Considering temporal and spatial changes of microbiota

The question of temporal and spatial structures in microbiomes is important for understanding of microbiome functioning in general. It is also of great significance for the understanding of specific processes, such as outbreaks of pathogens in biotechnological and food processing applications [90] as well as for predicting and controlling microbiomes [17]. In general, the size-diversity relationship initially described for macro-organisms was also evidenced for microbial communities in various ecosystems [91, 92].

**Fig 4:**
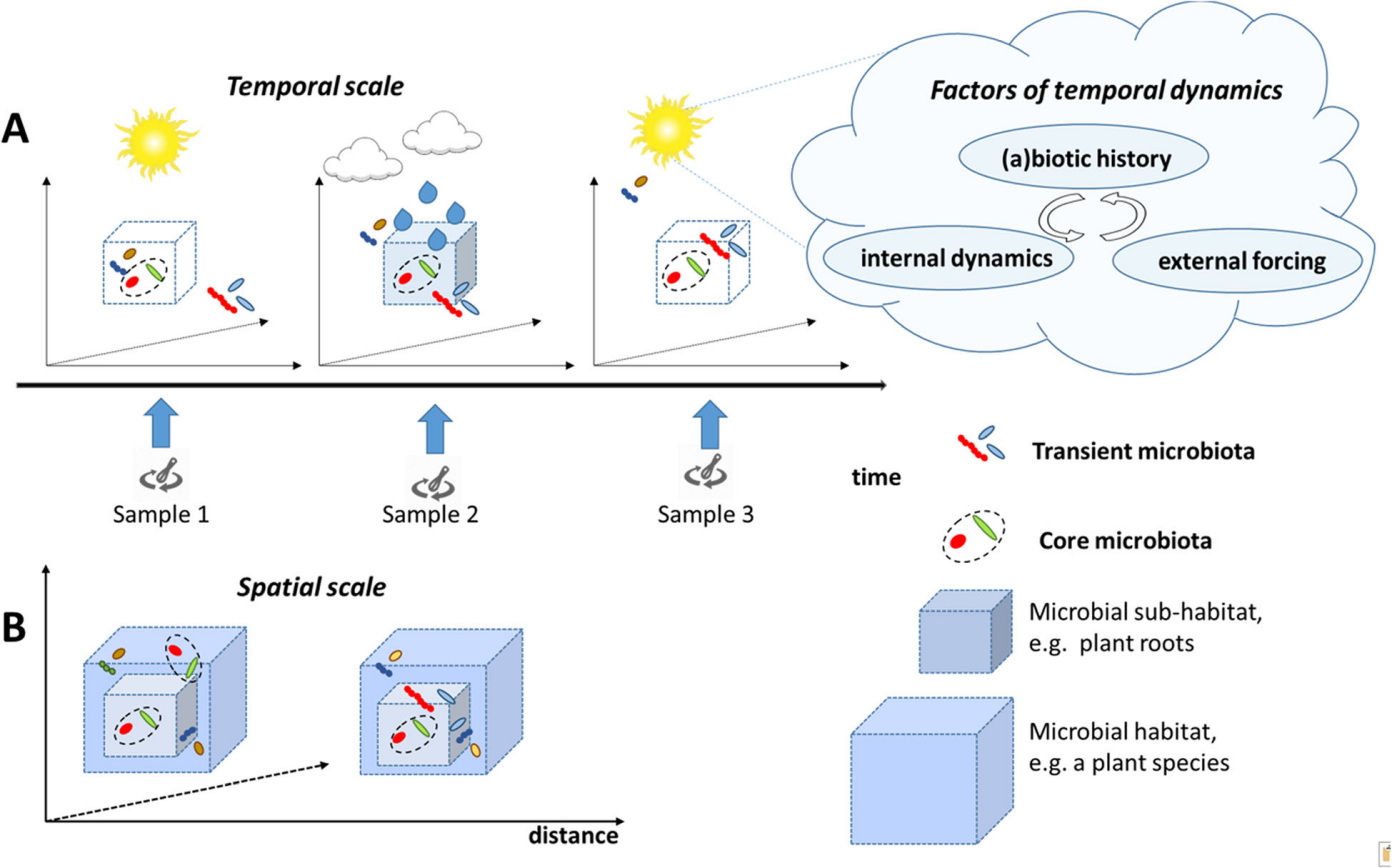
Microbiome dynamics in time and scale. **a** Temporal dynamics of the microbiome is characterized by considering both the transient state of microbiome as response to environmental perturbations, such as, for example, seasonal or circadian rhythms, and the resident state that contains rather constant core microbiota. **b** Spatial dynamics of the microbiome is characterized by variations in the microbial composition between similar habitats separated in space. The separation may be between the organisms (e.g., same plants species grown in two different locations), between the parts of one organism (e.g., plant roots and the whole plant), or even within an organ (e.g., comparing microbiomes of various intestinal segments)

Temporal dynamics within the microbiome can be assessed from the scale of seconds or minutes, reflecting the timespan of messenger RNA to the scale of centuries and millennia, during which microorganisms have coevolved with their host or within a particular environment [93]. Half-lives of bacterial mRNA depend on the genes transcribed but is usually in the range of minutes, while transcripts from archaeal genes are longer, and times of several hours have been reported [93, 94]. Importantly, although many authors in the past have linked microbial activity with the rRNA content, recent studies have indicated severe limitations of this concept, and only mRNA can be considered as a reliable indicator of the metabolic state [95]. Understanding the appropriate dimension of study across this range of temporal scales is critical for any microbiome manipulation, such as therapeutic strategies in human microbiome research or the use of biocontrol agents in the case of environmental studies. Careful consideration of the specific characteristics of the host of interest, such as circadian rhythms, seasonal variations, or growth stages in relation to the physiology of the host organism may help to identify optimal scales of assessment of temporal dynamics (Fig. 4a). Stegen et al. [17] suggest to consider three categories: (i) biotic and abiotic history, (ii) internal dynamics, and (iii) external forcing factors as factors influencing the temporal dynamics of microbiomes.

Most natural ecosystems are characterized by a high degree of spatial structuring, which has been considered to be important for many ecosystem services [96]. Considering spatial scale may imply comparison of microbial patterns between distant regions, as well as the less obvious differentiation between the subsists of the same habitats (Fig. 4b). Soils are primarily composed of micro-aggregates (< 0.25 mm), which bind soil organic carbon and protect it from removal by erosion and of macro-aggregates (0.25 to 2 mm), which limit oxygen diffusion and regulate water flow; each of the aggregates provides a unique ecological niche, with its characteristic microbiome structure [97]. In fact, it has been suggested that soils are the ecosystems with the most diverse composition of microbiota on Earth as a consequence of so many different niches being present at small spatial scales. A reduction of niches for instance, induced by tillage in agricultural practices, can result in a loss in microbial diversity [98]. As plants and tillage influence the development of soil structure to a large extent, a loss in plant diversity has a strong impact on the biodiversity of the soil microbiome too [99]. However, the answer to the question of “chicken or egg” (do changes in the soil microbiome induce shifts in the plant diversity or vice versa?) remains unclear. The colonization of hosts by microbiota is also not uniform. It is well known that, for example, leaves harbour a different microbiota compared to the root, and the root itself is heterogeneously colonized by microbes, with different microbiota along the length of the rhizosphere, and at the root surface vs. the root interior [100]. Recently, the topic of the seed microbiota has attracted attention as a possible mode for the vertical transmission of a core microbiota from one plant generation to another [101]. Similar to plants, the human body is not homogeneously colonized by microbes: each body compartment contains its own microbiota [102], and even microbiota from one body site may differ depending on the area of sampling (e.g., the skin microbiota [103]).

Microbial hotspots and hot moments are often closely interlinked. For example, soils are characterized by the existence of so-called microbial hotspots (spatial separation including the rhizosphere, drilosphere, or detritusphere) and hot moments (temporal dynamics). In hotspots, the fraction of actively metabolizing microorganisms is 2–20 times higher than in the non-hotspots [104], making temporal shifts in microbiome structure and function in hotspots much more dynamic compared to sites with less microbial activity.

### Defining the core microbiota

Based on co-occurrence analyses and experimental data capturing temporal and spatial dynamics of microbiomes, researchers have sought to define a core microbiota. This is indeed helpful as native microbiomes are often highly complex, comprising thousands of species across different kingdoms. Defining core microbiota can facilitate discrimination of the stable and permanent members of a microbiome from populations that may be intermittent, associated only with specific microbiome states, or restricted to specific environmental conditions. A first suggestion was provided by Shade and Handelsman [105], who defined a core microbiome as the suite of members shared among microbial consortia from similar habitats to identify the stable, consistent components across complex microbial assemblages. Currently, the core microbiota is mostly defined on the basis of DNA sequences with taxonomic information. Taking the resolution limits of DNA-based analyses (mainly amplicon sequencing of marker genes) into account, it is however obvious that core microbiomes have been predominantly defined using genus-level discrimination of a population, and strain-specific as well as functional variation was not considered. As a contrast, Lemanceau and coworkers [106] suggested a functional core microbiota that encompasses microbial vehicles carrying replicators (genes) with essential functions for holobiont fitness. Recently, Toju et al. [107] presented the concept of the core microbiota specifically for managing agricultural ecosystems into species-rich communities; they define the core as “sets of microorganisms that form cores of interactions that can be used to optimize microbial functions at the individual plant and ecosystem levels.”

Astudillo-García and coworkers [108] evaluated the impact of different core microbiota definitions when interrogating highly diverse microbial systems of marine sponges. While caution must be exercised when defining core communities, fortunately, overall results have appeared to be relatively insensitive to variations in core definitions. The transient microbiota changes over time, depending on the environmental conditions, availability of nutrition, and/or growth and health stage or even diurnal rhythms of hosts. In contrast, the “core” microbiota appears to remain fairly constant. In terms of temporal dynamics, the core microbiota describes the microbial community that is constantly associated with a given host genotype or a specific environment (Fig. 4a). Exceptions to this concept have also been described, e.g., in the microbiome optimally adapted to reoccurring hydration/dehydration cycles, different bacterial communities fulfill different functions within the cycle: both belong to the core [109]. Similarly, at a spatial scale, e.g., considering plants grown in the same field or in a range of soils of one geographical region [110], the core microbiota does not change (Fig. 4b).

### From functional predictions to the phenotype

**Fig. 5:**
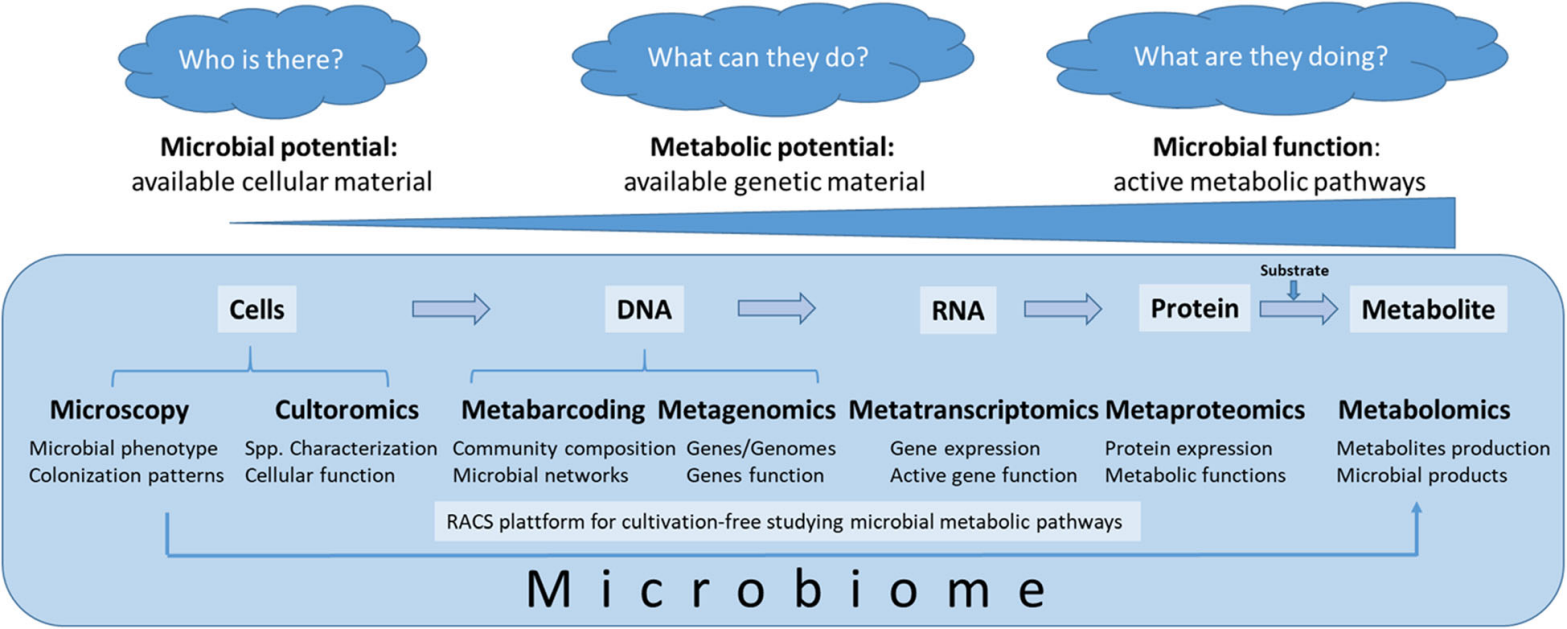
Methods for assessing microbial functioning. Complex microbiome studies cover various areas, starting from the level of complete microbial cells (microscopy, culturomics), followed by the DNA (single cell genomics, metabarcoding, metagenomics), RNA (metatranscriptomics), protein (metaproteomics), and metabolites (metabolomics). In that order, the focus of the studies shifts from the microbial potential (learning about available microbiota in the given habitat) over the metabolic potential (deciphering available genetic material) towards microbial functioning (e.g., the discovery of the active metabolic pathways)

Currently available methods for studying microbiomes, so-called multi-omics, range from high throughput isolation (culturomics) and visualization (microscopy), to targeting the taxonomic composition (metabarcoding), or addressing the metabolic potential (metabarcoding of functional genes, metagenomics) to analyze microbial activity (metatranscriptomics, metaproteomics, metabolomics) (Fig. 5). Based on metagenome data, microbial genomes can be reconstructed. While first metagenome-assembled genomes were reconstructed from environmental samples [111], in recent years, several thousands of bacterial genomes were binned without culturing the organisms behind. For example, 154,723 microbial genomes of the global human microbiome were recently reconstructed from 9,428 metagenomes [112]. Our understanding, however, is still significantly limited due to the missing links between the massive availability of microbiome DNA sequence data on the one hand and limited availability of microbial isolates needed to confirm metagenomic predictions of gene function on the other hand. Metagenome data provides a playground for new predictions, yet much more data is needed to strengthen the links between sequence and rigorous functional predictions. This becomes obvious when considering that the replacement of one single amino acid residue by another may lead to a radical functional change, resulting in an incorrect functional assignment to a given gene sequence [113]. Additionally, cultivation of new strains is needed to help identify the large fraction of unknown sequences obtained from metagenomics analyses, which for poorly studied ecosystems can be more than 70%. Depending on the applied method, even in well-studied microbiomes, 40–70% of the annotated genes in fully sequenced microbial genomes have no known or predicted function [114]. Moreover, current estimates predict that domains with unknown functions will outnumber families of known function very soon [115]. There is a clear need for more classical microbiology including the use of targeted mutants in combination with microbial biochemistry to cope with this challenge. Moreover, there is much more to gain from thorough functional characterization of already discovered protein families with unknown function(s) than from further extending the list of these families. While multiphasic approaches combining (extensive) cultivation and cultivation-independent analyses have been state-of-the-art in environmental and plant microbiology for a long time [116–118], this is not generally the case for medical microbiology. Recently, reference genomes and culture collections of the human gut microbiota were established by high-throughput culturomics [119–121].

Understanding prokaryotic functional diversity is highly challenging, as 85 out of the currently established 118 phyla have not had a single species described to date [121]. Finally, the number of prokaryotic phyla may reach hundreds, and archaeal ones are among the least studied. This issue needs to be addressed by gathering meaningful taxonomic and functional information for not-yet cultured prokaryotes [121]. The growing gap between the diversity of *Bacteria* and *Archaea* held in pure culture and those detected by molecular methods has led to the proposal to establish a formal nomenclature for not-yet cultured taxa, primarily based on sequence information [122, 123]. According to this proposal, the concept of Candidatus species would be extended to the groups of closely related genome sequences, and their names would be published following established rules of bacterial nomenclature. The suggested alterations of the International Code of Nomenclature of Prokaryotes raise concerns regarding (1) the reliability and stability of nomenclature, (2) the technological and conceptual limitations as well as availability of reference genomes, (3) the information content of in silico functional predictions, and (4) the recognition of evolutionary units of microbial diversity. These challenges need to be overcome to arrive at a meaningful taxonomy of not-yet cultured prokaryotes with, at present, poorly understood phenotypes [121]. Against this backdrop, significant efforts have been made to cultivate bacteria from diverse environments. Staley and Konopka identified in 1985 “the great plate count anomaly” which describes the fact that 90 to 99.9% of bacterial species cannot be grown under standard laboratory conditions [124]. For some micro-habitats, especially those with high nutrient content and microbial activity, the proportion of representative strains available in culture relative to the molecular species detected by sequencing grew from 35 to 65%, as it was stated for the gut microbiota [125]. Similar advances are needed for microbial populations from other natural habitats as well as for the eukaryotic members of the microbiome. Micro-eukaryotes, e.g., members of protozoa, fungi, and algae, can often be better cultivated and microscopically studied; however, their phylogeny and taxonomy are more complex and less studied. Interestingly, primer-free 16S and 18S rRNA gene sequencing from various environments has shown that among microeukaryotes there is a huge number of previously not detected taxa [126].

Apart from the in silico comparisons and cultivation methods currently used, a set of isotope-probing techniques is available to directly test functional hypotheses within complex microbial communities. These methods encompass DNA-, RNA-, protein-, and lipid-stable isotope probing (SIP) [127] as well as FISH-micro-autoradiography, FISH-NanoSIMS, and FISH-Raman micro-spectroscopy, with the latter three methods offering single cell resolution [128, 129]. Recently, a microfluidic Raman activated cell sorting (RACS) platform was developed [130]. In a recent study, Lee and coworkers allowed cells from mouse colon microbiota to metabolize an unlabeled compound of interest (mucin) in a presence of deuterated water [130]. Subsequently, the deuterium labeled cells that actively metabolized mucin were sorted out from the complex microbiomes using the RACS platform and further analyzed by the means of single-cell genomics and cultivation methods. This method allows linking microbial metabolic phenotypes to their genotypes in a novel cultivation-free way, and so makes it possible to process from the microbial potential directly to the microbial function (Fig. 5). Despite its advancements, the throughput of this functional sorting platform is still limited, and complementary novel technological solutions such as the combination of FISH and bioorthogonal noncanonical amino acid tagging (BONCAT) [131] will contribute to the more urgently required phenotype-centric studies in microbiome research.

### Host-microbe coevolution

**Fig. 6:**
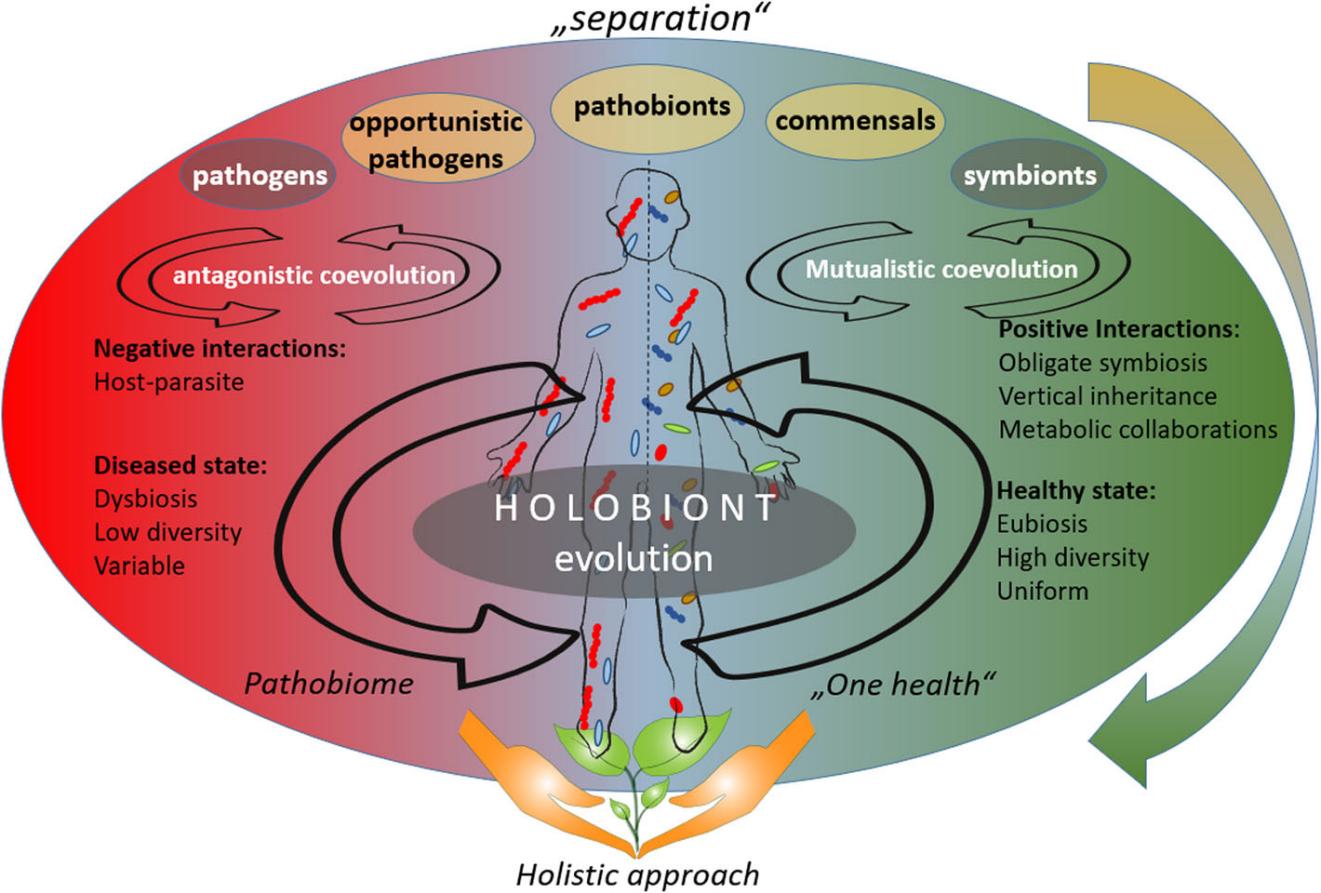
A shift in the understanding of the microbial-host coevolution from the “separation” theories to the holistic approach. The hosts and their associated microbiota are assumed to have coevolved with each other, whereby different approaches are considered to describe the coevolution theory. According to the “separation” approach (upper part of the figure), the microorganisms can be divided into pathogens, neutral, and symbionts, depending on their interaction with their host. The coevolution between host and its associated microbiota may be accordingly described as antagonistic (based on negative interactions) or mutualistic (based on positive interactions). The recent emerge in publications about opportunistic pathogens and pathobionts gave a shift towards holistic approach in the coevolutions theory (lower part of the figure). The holistic approach sees the host and its associated microbiota as one unit (so-called holobiont), that coevolves as one entity. According to the holistic approach, holobiont’s disease state is linked to dysbiosis, low diversity of the associated microbiota, and their variability: a so-called “pathobiome” state. The healthy state, on the other hand, is accompanied with eubiosis, high diversity, and uniformity of the respective microbiota. The dynamic flow of microorganisms from one host to another and to the environment, described by the One Health concept, underpins the holistic approach in the coevolution

The close relationships between hosts and their associated microorganisms gave rise to the theory of coevolution of the host and its associated microbiota [132, 133]. Coevolution is the reciprocal adaptation of lineages in response to one another [134]. An example is the establishment of early land plants that was facilitated by symbiotic fungal associations, suggesting that plants have coevolved with microbes since their first appearance on land [135]. Another example are eukaryotes themselves; mitochondria and plastids are organelles within eukaryotic cells that are derived from endosymbiotic bacteria and that, throughout coevolution, have become entirely dependent on their hosts and vice versa. Host-microbe coevolution is important to consider in order to facilitate a holistic understanding of the microbiota [12, 136] (Fig. 6).

The division of microorganisms into beneficial, pathogenic, and neutral based on microbial interactions with their hosts is a part of the widely cited microbiome definition by Lederberg and McCray [42]. According to their interpretation, antagonistic coevolution includes host-parasite interactions, while mutualistic coevolution is present when positive interactions prevail (Fig. 6). Such positive interactions may evolve towards obligate symbioses, vertical inheritance, and metabolic collaborations [137] (Fig. 6). Categorizing pathogenic, beneficial, and neutral microorganisms according to their interaction with their host may be useful for studies where microbe-host interactions play a central role in mediating host fitness, such as medical studies. The interpretation of the pathogenicity data, however, should be taken with caution [83]. Recent studies on opportunistic pathogens showed that host-microbe interactions depend not only on the host, but also on the entire microbiome [138, 139]. Naturally disease-suppressive soils suggest a similar situation for plants and many opportunistic soil-borne pathogens [23]. Furthermore, in many environmental studies, there may be no specific host available for extended periods of time, making the division into pathogens and beneficials irrelevant. Instead of looking into the interaction of one specific microorganism with its host, one can consider the holistic approach based on the holobiont theory (Fig. 6). Following this approach, the beneficial interplay of the host and its microbiome is responsible for maintaining the health of the holobiont, while diseases are often correlated with microbial dysbiosis [20, 140]. Within this context of dysbiosis, the “pathobiome” concept (Fig. 6), which represents the pathogenic agent integrated within its biotic environment, was established and applied to multiple pathosystems [141]. This approach suggests that microbial diversity is a key factor in preventing diseases in plants [23] as well as in the human gut [142]. Despite the numerous studies, defining a “healthy microbiota”, the borders between eubiosis and dysbiosis still remain a major challenge for the future [20]. Another interesting interpretation of microbial-host interactions is a so-called “Anna Karenina principle” [143]. It states that paralleling Leo Tolstoy’s dictum that “all happy families look alike; each unhappy family is unhappy in its own way,” dysbiotic individuals vary more in microbial community composition than healthy individuals. For example, the microbiomes of healthy corals were found to be more uniform than those of diseased corals [143]. Similarly, a very recent study found that community composition and immune responses are significantly less stable in individuals with inflammatory bowel disease than in healthy individuals [144].

Evolutionary processes and selection pressures significantly drive host-microbe interactions; therefore, differentiation into man-made, anthropocentric categories can change over time. One example is *Helicobacter pylori* that was the dominant microbe in the stomachs of almost all people in the early twentieth century and almost disappeared in just 100 years [22]. *H. pylori* is on one hand a risk factor for peptic ulcers and stomach cancer; the loss of this bacterium, on the other hand, is associated with asthma, hay fever, or skin allergies in childhood [145]. Another example of the complex evolutionary interactions between microbes and their prospective hosts is the emergence of pathogens like *Stenotrophomonas maltophilia,* a bacterium of plant-associated origin that is indistinguishable from the global opportunistic human-pathogenic strain [139, 146]. Altogether, natural selection is perceived to favor hosts that shape the community composition to promote a beneficial host-microbe symbiosis [147], yet many factors can unbalance this dynamic to induce selection for pathogenic or antagonistic microbes within the host microbiome. Based on computational models, Lewin-Epstein et al. [148] suggested that microbes that manipulate their hosts to act altruistically could be favored by selection and may play a role in the widespread occurrence of altruism.

Host-microbe coevolution leads to specific microbiomes associated with plants and animals [139, 149]. The extent of this specificity is influenced by many factors and varies between the phylogenetic branches. For mosses, which represent the oldest land plants on Earth, an extraordinarily high degree of plant specificity was described. This specificity was independent from geographical origin of the host and was vertically transmitted from the sporophyte to the gametophyte and vice versa [150, 151]. Domestication and breeding activities can also significantly shape host microbiomes and in some cases to a stronger extent than expected [152]. The theory of the disappearing microbiota suggests that prevalent chronic diseases are caused by the anthropogenic microbiome shifts towards reduced diversity [22]. Considering this, it is important to reconsider our activities (e.g., “overcleaning” of home environments) and evaluate crop management approaches, such as breeding strategies, to avoid the loss of co-evolved beneficial host-microbe interactions.

### Technical Standards in Microbiome Research

In the face of the pronounced and ongoing technical advances in microbiology over the last decade, the research community has failed to set consistent standards for microbiome research. This has resulted in many drawbacks, including missing possibilities for cross-comparison of data between labs or the implementation of data generated with “old” technologies in more recent studies.

A large amount of publications is available that show bias in DNA extraction and processing procedures for subsequent analysis of microbiomes [153]. The use of a defined mock community can on the one hand help to determine the best possible extraction method and on the other hand serve as an internal control to estimate the possible bias throughout the workflow and analysis. However, even the implementation of mock communities into the analysis cannot solve all issues, which introduce a bias into the molecular analysis of microbiomes form environmental samples. For example, natural sorption processes of soil microorganisms to soil particles cannot be mirrored by the application of mock communities to soil. Further, up to 80% of the extracted DNA in complex habitats like soils can contain relic DNA (extracellular DNA from the dead cells as well as excreted DNA from living cells). Such relic DNA can inflate the observed prokaryotic and fungal diversity and cause inaccurate estimation of taxon relative abundances [53]. A possible solution was presented by Nocker et al. [154] by using popidium monoazide (PMA) which only penetrates membrane-damaged cells, where a photo-induced azide group covalently binds to DNA; this cross-linking effectively inhibits PCR amplification of DNA from dead cells of both Gram-negative and Gram-positive bacteria. This procedure was successfully applied in microbiome studies, especially those with low microbial abundance and activity [155, 156], but it is not a standard yet. A well-known example in DNA processing is the selective amplification induced by primer selection and PCR or the large number of unknown reads when metagenomes are analyzed [152, 157, 158]. A primer-free rRNA gene sequencing approach developed by Karst et al. [126] facilitated the discovery of unknown microbial diversity. The authors suggest to combine poly(A)-tailing and reverse transcription of SSU rRNA molecules with synthetic long-read sequencing. Using this approach, they were able to generate high-quality, full-length SSU rRNA sequences, without primer bias, at high throughput and observed a large proportion of so far undescribed diversity in their study [126].

Bioinformatics analysis of obtained sequence data is neither standardized nor biases that are clearly expressed. For performing the data evaluations of 16S rRNA gene sequences, free software solutions and well-working forums like Qiime2 [159] and Mothur [160] are available. Operational taxonomic units (OTUs) are used for long time to analyze high-throughput barcoding, e.g., using marker-genes like 16S rRNA and or the ITS region for high throughput sequencing data. In addition, amplicon sequence variants (ASVs) can be used that based on exactly resolved, down to the level of single-nucleotide differences over the sequenced gene region. Benefits of finer resolution are immediately apparent, and the status of ASVs as consistent labels with intrinsic biological meaning identified independently from a reference database [154]. The improvements in reusability, reproducibility, and comprehensiveness are sufficiently great so that ASVs could replace OTUs as the standard unit of marker-gene analysis and reporting [161]. Several studies and joint projects have been performed to compare different tools and pipelines, showing significant differences in outcome depending on the tool or pipeline and the settings used [162–165]. It is obvious that besides sufficient sequencing depth, the choice of database for annotation of reads has a strong influence on the outcome. Moreover, the choice of parameters such as cutoff values, filtering of chimeras, and other, which is often done automatically, has a strong influence on the outcome. For example, as a result of data evaluation methods allowing investigation of taxa with a representation of more than 1% only, rare taxa are often neglected [166]. Such rare taxa, however, constitute up to 28% of all microbes, can represent key players in some habitats, and may be of importance for structuring communities [167]. They can have an over-proportional role in biogeochemical cycles and may be a hidden driver of microbiome function [168–170]. Moreover, microorganisms carrying antibiotic resistance genes often belong to the rare taxa in native microbiomes; under stress conditions, they provide the insurance for health and survival and ensure the plasticity of the ecosystem [171]. However, in the animal and human gut microbiome, these aspects can be crucial for outbreaks and therapies and are therefore implemented in the WHO forecast of spread in antibiotic resistance. Here, also, computing resources are still an issue making bioinformatics the bottleneck of microbiome analysis. Ten Hoopen and colleagues described that, for example, the subset of the TARA Oceans Microbiome Project that has been size-fractioned for prokaryotes comprises 135 samples. For the analysis of these samples, 248 runs containing 28.8 billion reads with an analysis output representing about 10 TB of data were necessary. The extensive study resulted in 23.2 billion predicted protein coding sequences [172]. In an attempt to deal with such big data, several global microbiome projects such as Human Microbiome Project [44] and Earth Microbiome Project [173] emerged in the past 10 years.

Further large gaps in metadata that limit microbiome analysis by impeding the comparability and integrative analysis of studies are obvious. There are indeed some repositories for data, e.g., the sequence read archive SRA (NCBI [174]), the European Nucleotide Archive (ENA [175]), or the CNGB Nucleotide Sequence Archive (CNSA [176]). However, in many cases, data are “available on request” only, which contradicts Findable, Accessible, Interoperable, and Reusable (FAIR) principles [177]. Even if data are available, the metadata often lack important information and are not reviewed sufficiently by the repositories including information on the experimental and statistical design of a study. Schloss and colleagues published a comprehensive review, about identification and overcoming threats to reproducibility, replicability, robustness, and generalizability in microbiome research [178]. Thus, there is an urgent need of standardization and the development of platforms for correct and comprehensive metadata repositories, as shown by Proctor [2] who developed a Project and Sample Application Standard for Human Pathogen/Vector Genomic Sequences. Ten Hoopen and colleagues [172] described a well-designed strategy that allows to set and apply standards and to get comparable and reusable data from microbiome research, following the FAIR principle.

## Future perspectives and challenges for microbiome research

**Fig. 7:**
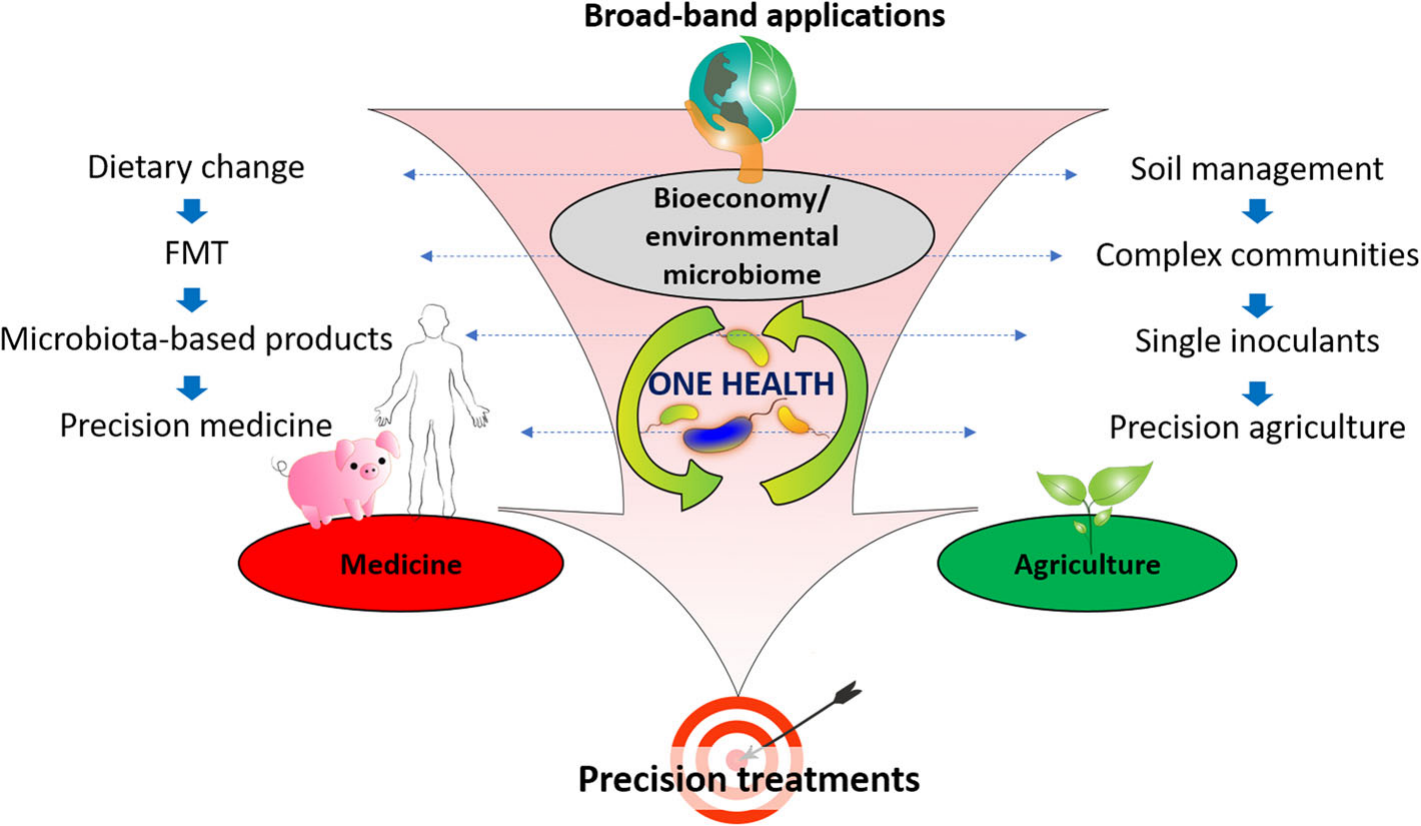
The schematic showing the cross-field microbiome-application trend that goes from broad-band applications direction microbiome-based precision treatment in all areas of microbiome research, such as agriculture, human and animal medicine, and bioeconomy, while the interconnection between these areas by the means of the cycling of subsets of microbial communities is an underlying concept behind the One Health approach. The synergies between the microbiome applications in the areas of medicine (left) and agriculture (right) are shown with the horizontal arrows following the flow (vertical arrows) from the broadband applications (upper part) to the precision treatments (lower part)

The increasing availability of microbiome data driven by advances in -omics technologies has led to dramatic increases in our understanding of the potential for microbiomes to enhance productivity and sustainability of diverse systems [6, 179, 180]. The grand vision of applied microbiome research is to improve health of humans, animals, plants, and whole ecosystems. In general, microbiomes can be managed either directly by applying (i) microbiome transplants, (ii) microbes with beneficial properties, or (iii) microbiota-active metabolites, or indirectly by changing environmental conditions in a way that microbiomes also shift their structure and function from dysbiosis into a healthy state [180, 181]. When comparing microbiome-based applications across humans, animals, and cropping systems, a striking synergy is visible (Fig. 7). Although the respective fields are not yet well connected, a consistent trend has become evident in all areas. This trend involves a focus on tailored treatments, such as for example, “next-generation” precision farming or personalized medicine (Fig. 7). This concept recognizes that not all individual hosts and their associated indigenous microbiomes will respond in the same way to a particular introduced microbe, microbiome transplant, or metabolite. Instead, it relies upon fundamental understanding of those particular host-microbe, environment-microbe, and microbe-microbe interactions that mediate microbiome assembly and functional capacities across diverse settings. Stegen et al. [17] suggest in their conceptual framework for microbiome management greater crosstalk across different areas, e.g., leveraging-specific ecological concepts from environmental microbiome science to guide optimization of strategies to manipulate human microbiomes towards improved health.

The human microbiome is emerging as a key target of personalized medicine by offering interesting solutions for a variety of environmental and metabolic diseases [182, 183]. In this respect, the human microbiome not only contributes to inter-individual variability in all aspects of a disease, but also represents a potential target for management, which could be modulated by therapeutics, dietary changes, the use of pre-, pro- or synbiotics, life biotherapeutic products corresponding to given species or mix of species (synthetic microbiota), and microbiome transplants [184–187]. Fecal microbiota transplantation is an approved treatment against recurrent and refractory *Clostridioides difficile* infection [188, 189] in the USA. Despite the mechanisms behind its efficacy that remain largely unknown, its applications in recent years rapidly expanded beyond gastrointestinal disorders to multiple fields such as a potential therapy for obesity, metabolic syndrome, and liver diseases [190–192]. Microbial heterogeneity between humans and across spatial and temporal gradients requires multidimensional datasets and a unifying set of theories and statistical tools to analyze the human microbiome and fully realize the potential for microbiome-based therapeutics [193]. Overall, the importance of a healthy gut microbiome is also highlighted by the recently described gut—brain, gut-liver, and gut—lung axis, making the gut a central organ for human health [194–196].

In analogy, the plant microbiome was identified as a key for the next green revolution (Science Breakthroughs by 2030 [197]). A systems approach integrating plant breeding, precision farming, agricultural management, and microbiome research provides a powerful strategy for improving sustainable crop production in a changing world [7, 148, 198]. Such an approach that encompasses many biological and geophysical components that may affect a plant's production in a specific environment was established by an interdisciplinary team as “Phytobiomes Roadmap” [199]. The species- and habitat-specific plant microbiota contributes multiple aspects to the functioning of the plant holobiont, such as (i) seed germination and growth, (ii) nutrient supply, (iii) resistance against biotic and abiotic stress factors, and (iv) production of bioactive metabolites [4]. Due to its importance for crop health, the plant microbiome has been studied for a long time. Moreover, an extensive list of microbiome management strategies and products was developed in agriculture including (i) microbiome transplants (straw dung and biodynamic additives), (ii) microbial inoculants, (iii) microbial and plant extracts, and (iv) methods to change environmental conditions [181]. In the last decades, management-intensive agriculture has relied predominantly on synthetic chemicals and has resulted in serious environmental and health problems as well as biodiversity losses [200]. Research on plant microbiomes, on the other hand, will support targeted and predictive management approaches that are suited to the specific conditions of the field and can thus result in greater sustainability. Similar to the human gut microbiome, the seed/rhizosphere microbiome is crucial for the plant, and the seed is a perfect target for next-generation microbials [100, 201]. Pérez-Jaramillo and colleagues [152] proposed the “back to the roots” approach for seeds, which offers an interesting opportunity for unraveling the seed microbiomes of wild relatives and ancient heirloom breeds of crop cultivars to save beneficial seed microbiomes for agriculture. Harnessing seed microbiomes of wild relatives of crop plants or from promising biological resources potentially enables a matching symbiosis between the plant and its specific seed microbiota [202]. The post-harvest microbiota is closely linked with our food microbiota, which can also be managed for desired functional properties of food products regarding, safety and preservation issues, organoleptic, or health properties. This represents a relative yet unexplored microbiome-based application that is benefiting of the emerging amount of data on food ecosystems [203].

The importance of microbiomes goes beyond the health of individual hosts. Microbes from different hosts and ecosystems can strongly interact and influence one other [204, 205]. These observations have led to the slogan “A healthy environment promotes healthy humans” which endorsed the “One Health concept.” According to the World Health Organization, “One Health” is an approach designing and implementing programs, policies, legislation, and research in which multiple sectors communicate and work together to achieve better public health outcomes. An expansion of the One Health concept including environmental health and its relation to human cultures and habits suggests that the lifestyle-microbiota-human health connection should be taken into account in societal decisions and policy making [206] (Fig. 7). For example, urbanization is associated with increases in allergies, asthma, and other chronic diseases. Besides, overall pollution pattern, a significant loss of microbial diversity, has been observed in urban areas, which has been associated with disease development [206]. Dominguez-Bello et al. [16] suggest that changes in the human microbiota occurring concomitantly with industrialization may be the underlying factor dramatic increases in metabolic, immune, and cognitive diseases, including obesity, diabetes, asthma, allergies, inflammatory bowel disease, and autism in the “developed” world. The loss of diversity in turn is correlated with an increase in bacterial resistance against antibiotics, thereby indicating a need for implementing strategies to restore bacterial diversity in built environments [156]. Understanding the complex connections among microbiomes across diverse hosts and habitats and their relationships with the health of the humans, animals, and plants, opens the potential for innovative and holistic approaches to diagnosis, treatment, and intervention within the context of the One Health concept [3, 23, 197, 206]. In analogy to One Health, different concepts exist linking human with environmental health; the planetary health concept is the most popular one [207]. This topic is also acknowledged in a number of national and international strategies for bioeconomy, where a sustainable, biological production of goods meets the demands of the economy. Without doubt to make these concepts a story of success, it requires interlinked strategies not only between different disciplines of natural science but beyond and integration of social sciences as well as stakeholders.

However, what we are facing nowadays is often the opposite: biodiversity loss, pollution, ozone depletion, climate change, and crossing of biogeochemical cycle boundary are the anthropogenic factors characterizing our epoch, the Anthropocene. The Antropocene is also reflected in the planetary boundary concept [208]. Four of nine planetary boundaries have now been crossed as a result of human activity: climate change, loss of biosphere integrity, land-system change, and altered biogeochemical cycles. First, studies indicate that they change functional and genetic diversity of the entire microbiota; however, more knowledge on the impact of these anthropogenic factors on different microbiomes and their consequences for our planet is definitively necessary [11]. Here, we recognize urgent need for more research, especially on environmental microbiomes and mechanistic insights in anthropogenic-driven changes. Understanding marine and terrestrial microbiomes and their interplay could be definitively a key to finding solutions for these massive challenges associated with the Antropocene. Microbiome management and the development of high-potential microbiome-based innovations are promising for various application fields but should go along with the careful assessment of the putative environmental impact of these new and promising technologies.

## Conclusions

Based on the recent advances in the area, we suggest the revival of the original definition of the microbiome term suggested by Whipps et al. [40]. The definition, which contains all important points that are valid even 30 years after its publication in 1988, was extended by two explanatory sentences differentiating the terms microbiome and microbiota and pronouncing its dynamic character.

The microbiome is defined as a characteristic microbial community occupying a reasonable well-defined habitat which has distinct physio-chemical properties. The microbiome not only refers to the microorganisms involved but also encompass their theatre of activity, which results in the formation of specific ecological niches. The microbiome, which forms a dynamic and interactive micro-ecosystem prone to change in time and scale, is integrated in macro-ecosystems including eukaryotic hosts, and here crucial for their functioning and health.

The microbiota consists of the assembly of microorganisms belonging to different kingdoms (Prokaryotes [Bacteria, Archaea], Eukaryotes [e.g., Protozoa, Fungi, and Algae]), while “their theatre of activity” includes microbial structures, metabolites, mobile genetic elements (e.g., transposons, phages, and viruses), and relic DNA embedded in the environmental conditions of the habitat.

Furthermore, we consider the following points as crucial for microbiome studies:

1. The core microbiota is a suite of members shared among microbial consortia from similar habitats, which is important for understanding stability, plasticity, and functioning across complex microbial assemblages.
2. Theories adapted from macro-ecology might be helpful to understand patterns of microbiome dynamics in different environments, but their general application needs to be verified.
3. An appropriate experimental, methodological, and statistical design is the basis for each microbiome study. The spatial, temporal, and developmental integration should be implemented in the design by (i) choosing the appropriate sampling frequencies based on the system characteristic in order to capture complete core and transient microbiota, (ii) considering the appropriate spatial scale of the system by recognizing that also subsets on scales are relevant for microbiota assessment, and (iii) for strongly dynamic systems, investigation of a space-time continuum of the microbial distribution instead of capturing one particular moment/space unit should be considered.
4. Microbiome research is strongly driven by methodological advances. Despite all progress in this area, there is no perfect and universal method. A toolbox of technologies will reduce bias resulting from each individual technology and result in a more complete view on the biological system as a whole.
5. Microbial functions play an important role in the ecosystem. Therefore, we recommend including a combination of several currently available methods in microbiome studies that allow deeper insights into microbial functioning.
6. Despite large amounts of –omics data, produced in the last decade, we still lack information on the organisms behind. Thus, strong efforts are needed for implementing more cultivation-based approaches into microbiome research, which allows a description of ecotypes and modes of adaptation of specific microbial groups to their environments.
7. Microbial interactions are the basis for functioning and evolutionary dynamics of microbial communities. Therefore, we advocate considering interactions in the study design.
8. Host-microbe interactions shape the reciprocal fitness, phenotype, and metabolisms, giving raise to the theory of coevolution of microbiota and their host [209]. We suggest a holistic approach based on the hologenome theory of evolution. Disease state of the holobiont is characterized by dysbiosis (pathobiome), while eubiosis refers to a balanced host-microbe interaction (“healthy” microbiome).
9. The division of microorganisms into beneficial, pathogenic, and neutral according to microbial interactions with their hosts is based on an anthropocentric view [210]. Indeed, the physiology of the host and the whole microbiome substantially influence the outcome of the interaction.

Application of these clarifications and recommendations should assist researchers in designing their microbiome studies in a holistic way, which will help to develop microbial models and predictions, which in turn will accelerate our ability to design applications in in all areas of life.

## Supplementary information

**Additional file 1.**

## Data Availability

Not applicable.

## Abbreviations

BONCAT: Bioorthogonal noncanonical amino acid tagging
CNSA: CNGB Nucleotide Sequence Archive
DIET: Direct Interspecies Electron Transfer
ENA: European Nucleotide Archive
(FAIR) principles: Findable, Accessible, Interoperable, and Reusable
FISH-CLSM: Fluorescence in situ Hybridization and Confocal Laser Scanning Microscopy
HGT: Horizontal gene transfer
ITS: Internal transcribed spacer
RACS: Raman activated cell sorting
SIP: Lipid-stable isotope probing
SRA: Sequence read archive
