# Correction to: Microbiome definition re-visited: old concepts and new challenges

**DOI:** 10.1186/s40168-020-00905-x

**Published:** 2020-08-20

**Authors:** Gabriele Berg, Daria Rybakova, Doreen Fischer, Tomislav Cernava, Marie-Christine Champomier Vergès, Trevor Charles, Xiaoyulong Chen, Luca Cocolin, Kellye Eversole, Gema Herrero Corral, Maria Kazou, Linda Kinkel, Lene Lange, Nelson Lima, Alexander Loy, James A. Macklin, Emmanuelle Maguin, Tim Mauchline, Ryan McClure, Birgit Mitter, Matthew Ryan, Inga Sarand, Hauke Smidt, Bettina Schelkle, Hugo Roume, G. Seghal Kiran, Joseph Selvin, Rafael Soares Correa de Souza, Leo van Overbeek, Brajesh K. Singh, Michael Wagner, Aaron Walsh, Angela Sessitsch, Michael Schloter

**Affiliations:** 1 Institute of Environmental Biotechnology, Graz University of Technology, Graz, Austria; 2 Helmholtz Zentrum München, Oberschleissheim, Germany; 3 MICALIS, INRA, AgroParisTech, Université Paris-Saclay, 78350 Jouy-en-Josas, France; 4 Waterloo Centre for Microbial Research, University of Waterloo, 200 University Avenue West, Waterloo, ON N2L 3G1 Canada; 5 Metagenom Bio, 550 Parkside Drive, Unit A9, Waterloo, ON N2L 5 V4 Canada; 6 Guizhou Provincial Key Laboratory for Agricultural Pest Management of the Mountainous Region, Guizhou University, Guiyang, 550025 Guizhou China; 7 Department of Agricultural, Forest and Food Sciences, University of Turin, Turin, Italy; 8 International Alliance for Phytobiomes Research, Lee’s Summit, MO USA; 9 MICA, INRA, 78350 Jouy-en-Josas, France; 10 Laboratory of Dairy Research, Department of Food Science and Human Nutrition, Agricultural University of Athens, Athens, Greece; 11 Department of Plant Pathology, University of Minnesota, St. Paul, MN 55108 USA; 12 BioEconomy, Research, & Advisory, Valby, Denmark; 13 CEB-Centre of Biological Engineering, University of Minho, Campus de Gualtar, 4710-057 Braga, Portugal; 14 Department of Microbial Ecology and Ecosystem Science, University of Vienna, Vienna, Austria; 15 Agriculture and Agri-Food Canada, Ottawa, Canada; 16 Sustainable Agriculture Sciences, Rothamsted Research, Harpenden, UK; 17 Biological Sciences Division, Pacific Northwest National Laboratory, Richland, WA 99352 USA; 18 Bioresources Unit, AIT Austrian Institute of Technology, Tulln, Austria; 19 CABI, Bakeham Lane, Egham, Surrey TW20 9TY UK; 20 Department of Chemistry and Biotechnology, Tallinn University of Technology, Tallinn, Estonia; 21 Laboratory of Microbiology, Wageningen University & Research, Wageningen, the Netherlands; 22 MGP, INRA, 78350 Jouy-en-Josas, France; 23 Dept of Food Science and Technology, Pondicherry University, Puducherry, India; 24 Department of Microbiology, Pondicherry University, Puducherry, India; 25 Genomics for Climate Change Research Center (GCCRC), Universidade Estadual de Campinas (UNICAMP), Campinas, SP Brazil; 26 Hawkesbury Institute for the Environment, Western Sydney University, Penrith, NSW Australia; 27 Global Centre for Land-Based Innovation, Western Sydney University, Penrith, NSW Australia; 28 Teagasc Food Research Centre, Moorepark, Fermoy, Co. Cork, Ireland

**Correction to: Microbiome 8, 103 (2020)**

**https://doi.org/10.1186/s40168-020-00875-0**

Following publication of the original article [1], an error was identified in the affiliation of the 8th author, Luca Cocolin.

The incorrect affiliation is: European Food Information Council, Brussels, Belgium

The correct affiliation is: Department of Agricultural, Forest and Food Sciences, University of Turin, Italy

The affiliation has been updated in this correction article.
