# Supplementary material for: Microbiome definition re-visited: old concepts and new challenges
Source: Microbiome. 2020 Jun 30;8:103. doi: 10.1186/s40168-020-00875-0 (PMC7329523; doi:10.1186/s40168-020-00875-0)
Supplement: Supplementary file 2 — Additional file 1. [file 40168_2020_875_MOESM1_ESM.docx]

**Supplemental File 1**

**1**. Lane N. The unseen world: reflections on Leeuwenhoek (1677) ‘Concerning little animals.’ Philos Trans R Soc B Biol Sci. 2015;370.

**2**. Dubey RC. Advanced Biotechnology. S. Chand & Company P Ltd, New Delhi-44 (India); 2014.

**3**. Heimstädt O. Das Fluoreszenzmikroskop. Zs F Wiss Mikrosk U F Mikrosk Tech. 1911;330–7.

**4**. Borman S, Russell H, Siuzdak G. A Mass Spec Timeline. Developing techniques to measure mass has been a Nobel pursuit. 2003;3.

**5**. Ruska E. The development of the electron microscope and of electron microscopy (Nobel Lecture). Angew Chem Int Ed Engl. Wiley Online Library; 1987;26:595–605.

**6**. Von Ardenne M. Das elektronen-rastermikroskop. Z Für Phys. Springer; 1938;109:553–72.

**7**. Fahimi HD. Perfusion and immersion fixation of rat liver with glutaraldehyde. Lab Investig J Tech Methods Pathol. 1967;16:736–50.

**8**. McDonald P. Waters Corporation: Fifty Years of Innovation in Analysis and Purification. https://www.sciencehistory.org/distillations/waters-corporation-fifty-years-of-innovation-in-analysis-and-purification. Accessed 28. May 2020.

**9**. Grunstein M, Hogness DS. Colony hybridization: a method for the isolation of cloned DNAs that contain a specific gene. Proc Natl Acad Sci. National Acad Sciences; 1975;72:3961–5.

**10**. Sanger F, Nicklen S, Coulson AR. DNA sequencing with chain-terminating inhibitors. Proc Natl Acad Sci U S A. 1977;74:5463–7.

**11**. Mullis KB. The unusual origin of the polymerase chain reaction. Sci Am. 1990;262:56–61, 64–5.

**12**. Bentley Lawrence J, Villnave CA, Singer RH. Sensitive, high-resolution chromatin and chromosome mapping in situ: Presence and orientation of two closely integrated copies of EBV in a lymphoma line. Cell. 1988;52:51–61.

**13**. Higuchi R, Fockler C, Dollinger G, Watson R. Kinetic PCR analysis: real-time monitoring of DNA amplification reactions. Biotechnol Nat Publ Co. 1993;11:1026–30.

**14**. Amann RI, Ludwig W, Schleifer KH. Phylogenetic identification and *in situ* detection of individual microbial cells without cultivation. Microbiol Rev. 1995;59:143–69.

**15**. Kulski JK. Next-generation sequencing—an overview of the history, tools, and “Omic” applications. Gener Seq Appl Chall. InTech, Rijeka, Croatia; 2016;3–60.

**16**. Mehrota RS, Aneja KR. An introduction to mycology. New Delhi, India: Wiley Eastern Ltd.; 1990.

**17**. Riedel S. Edward Jenner and the history of smallpox and vaccination. Proc Bayl Univ Med Cent. 2005;18:21–5.

**18**. Pasteur L. Mémoire sur la fermentation appelée lactique (Extrait par l’auteur). Mol Med. 1995;1:599.

**19**. Cowan ST. Heretical Taxonomy for Bacteriologists. Microbiology. 1970;61:145–54.

**20**. Evans AS. Causation and Disease: The Henle-Koch Postulates Revisited. Yale J Biol Med. 1976;49:175–95.

**21**. Waksman SA. Sergei Nikolaevitch Winogradsky: 1856-1953. Science. American Association for the Advancement of Science; 1953;118:36–7.

**22**. Dworkin M. Sergei Winogradsky: a founder of modern microbiology and the first microbial ecologist. FEMS Microbiol Rev. 2012;36:364–79.

**23**. Hartmann A, Rothballer M, Schmid M. Lorenz Hiltner, a pioneer in rhizosphere microbial ecology and soil bacteriology research. Plant Soil. 2008;312:7–14.

**24**. Griffith Fred. The Significance of Pneumococcal Types. J Hyg (Lond). 1928;27:113–59.

**25**. American Chemical Society. Alexander Fleming Discovery and Development of Penicillin - Landmark Am. Chem. Soc. https://www.acs.org/content/acs/en/education/whatischemistry/landmarks/flemingpenicillin.html- Accessed 28 May 2020.

**26**. Deichmann U. Early responses to Avery et al.’s paper on DNA as hereditary material. Hist Stud Phys Biol Sci. 2004;34:207–32.

**27**. Griffiths AJ, Miller JH, Suzuki DT, Lewontin RC, Gelbart WM. Bacterial conjugation. Introd Genet Anal 7th Ed. WH Freeman; 2000.

**28**. Crick F. Central Dogma of Molecular Biology. Nature. 1970;227:561.

**29**. Woese CR, Fox GE. Phylogenetic structure of the prokaryotic domain: the primary kingdoms. Proc Natl Acad Sci U S A. 1977;74:5088–90.

**30**. Prusiner SB. Novel proteinaceous infectious particles cause scrapie. Science. 1982;216:136–44.

**31**. Margulis L, Fester R, editors. Symbiosis as a source of evolutionary innovation: speciation and morphogenesis. Cambridge, Mass: MIT Press; 1991.

**32**. Flemming H-C. Biofilms and Environmental Protection. Water Sci Technol. 1993;27:1–10.

**33**. Fleischmann RD, Adams MD, White O, Clayton RA, Kirkness EF, Kerlavage AR, et al. Whole-genome random sequencing and assembly of Haemophilus influenzae Rd. Science. 1995;269:496–512.

**34**. Peterson J, Garges S, Giovanni M, McInnes P, Wang L, Schloss JA, et al. The NIH Human Microbiome Project. Genome Res. 2009;19:2317–23.

**35**. Vogel TM, Simonet P, Jansson JK, Hirsch PR, Tiedje JM, van Elsas JD, et al. TerraGenome: a consortium for the sequencing of a soil metagenome. Nat Rev Microbiol. 2009;7:252.

**36**. Gilbert JA, Meyer F, Jansson J, Gordon J, Pace N, Tiedje J, et al. The Earth Microbiome Project: Meeting report of the “1st EMP meeting on sample selection and acquisition” at Argonne National Laboratory October 6th 2010. Stand Genomic Sci. 2010;3:249–53.
